# Impacts of gestational age uncertainty in estimating associations between preterm birth and ambient air pollution

**DOI:** 10.1097/EE9.0000000000000031

**Published:** 2018-12-12

**Authors:** Benjamin E. Nealy, Joshua L. Warren, Matthew J. Strickland, Lyndsey A. Darrow, Howard H. Chang

**Affiliations:** a Department of Biostatistics and Bioinformatics, Emory University; b Department of Biostatistics, Yale University; c School of Community Health Sciences, University of Nevada, Reno.

## Abstract

**Background:**

Previous epidemiologic studies utilizing birth records have shown heterogeneous associations between air pollution exposure during pregnancy and the risk of preterm birth (PTB, gestational age <37 weeks). Uncertainty in gestational age at birth may contribute to this heterogeneity.

**Methods:**

We first examined disagreement between clinical and last menstrual period-based (LMP) determination of PTB from individual-level birth certificate data for the 20-county Atlanta metropolitan area during 2002 to 2006. We then estimated associations between five trimester-averaged pollutant exposures and PTB, defined using various methods based on the clinical or LMP gestational age. Finally, using a multiple imputation approach, we incorporated uncertainty in gestational age to quantify the impact of this variability on associations between pollutant exposures and PTB.

**Results:**

Odds ratios (OR) were most elevated when a more stringent definition of PTB was used. For example, defining PTB only when LMP and clinical diagnoses agree yielded an OR of 1.09 (95% confidence interval [CI] = 1.04, 1.14) per interquartile range increase in first trimester carbon monoxide exposure versus an OR of 1.04 (95% CI = 1.01, 1.08) when PTB was defined as either an LMP or clinical diagnosis. Accounting for outcome uncertainty resulted in wider CIs—between 7.4% and 43.8% wider than those assuming the PTB outcome is without error.

**Conclusions:**

Despite discrepancies in PTB derived using either the clinical or LMP gestational age estimates, our analyses demonstrated robust positive associations between PTB and ambient air pollution exposures even when gestational age uncertainty is present.

## Abstract 2

Supplemental Digital Content is available in the text.

**What this study adds**

A large number of studies have utilized birth records to examine associations between preterm birth and gestational exposure to ambient air pollution. One main criticism of these studies is that reported gestational age is subject to error, potentially resulting in outcome misclassification. Using birth records that contain gestational age estimated using both the last menstrual period and clinical assessment, this study finds that the positive associations between preterm birth and air pollution exposures are robust against uncertainty in gestational age. Moreover, estimated associations were most elevated when a more stringent definition of preterm birth was used.

Preterm birth (PTB), defined as gestational age (GA) less than 37 completed weeks, is a known predictor of increased infant mortality and morbidity, as well as long-term health consequences.^1–5^ While numerous epidemiologic studies have found positive associations between PTB and maternal exposure to ambient air pollution, recent systematic reviews and meta analyses have reported substantial heterogeneity in associations across studies.^6–8^ The most recent US Environmental Protection Agency Integrated Science Assessment concluded that relationships between air pollution and reproductive outcomes were “suggestive of a causal relationship.”^9–11^

In most previous studies, associations between ambient air pollution exposure and PTB were investigated by retrospectively linking live birth certificates and exposures based on maternal residential address. Compared to prospective birth cohorts, the use of birth records is cost-effective for acquiring sufficient sample size with large spatial-temporal coverage to estimate small but public health-relevant associations at the population level. Limitations of using birth records are well recognized, including bias in response (e.g., under-reporting of maternal alcohol and cigarette use^12,13^), lack of important confounders (e.g., diet, physical activity, and body mass index), and random recording errors. For PTB studies, uncertainty in GA leads to several unique challenges.^14^ First, uncertainty in GA can lead to outcome misclassification, particularly around the 37-week cutoff. Second, GA is used to back-calculate conception date and construct the exposure profile during pregnancy.

In the United States after 2000, birth certificates provide two sources of information on GA, and both sources are subject to errors. The first estimate uses the reported date of the last menstrual period (LMP), which may suffer from recall errors and inter-individual variability in timing between LMP and conception.^15^ A second clinical estimate is based on a combination of various clinical measurements and physician judgment. However, accuracy can depend on whether these measurements are based on newborn assessment or prenatal ultrasounds and on the quality of the clinical examination.^16^ Some work has been done comparing clinical estimates and estimates of GA from birth certificates, often showing only moderate concordance.^17–20^ Previous studies of air pollution and PTB have utilized GA defined a priori by the investigators using either LMP^21–23^ or the clinical estimate.^14–26^ Often, when the preferred source of GA information is missing, the other GA estimate is used.

Few studies have evaluated effects of uncertainty in GA estimates when examining associations with ambient air pollutant exposure or consider the use of different GA estimates as a sensitivity analysis. Recently, Rappazzo et al.^27^ found that results can be sensitive to using clinical or LMP GA estimates in an analysis of fine particulate matter and PTB in New Jersey, Pennsylvania, and Ohio, United States. In this study, we evaluated the impact of GA definitions on air pollution risk associations using birth certificates in Atlanta, Georgia, between 2002 and 2006. We expand the work of Rappazzo et al. by considering additional GA definitions and ambient air pollutants, and we implement a multiple imputation approach to incorporate GA uncertainty in analyses.

## Methods

### Health and air quality data

We obtained individual-level birth certificate data for the 20-county Atlanta metropolitan area (Barrow, Bartow, Carroll, Cherokee, Clayton, Cobb, Coweta, DeKalb, Douglas, Fayette, Forsyth, Fulton, Gwinnett, Henry, Newton, Paulding, Pickens, Rockdale, Spalding, and Walton counties) from the Office of Health Indicators for Planning, Georgia Department of Public Health. Georgia birth certificates recorded two estimates of GA in complete weeks: a clinical estimate and an LMP-based estimate. GA estimates were used to back-calculate conception date, which was assumed to occur at the second gestational week based on obstetric convention. We included singleton pregnancies with conception dates between January 1, 2002, and February 28, 2006, to avoid the fixed-cohort bias (n = 587,937).^28,29^ Additional exclusion criteria included (1) maternal residential address at delivery unsuccessfully geocoded to the 2000 Census block group (n = 12,562), (2) birth weight less than 400 g (n = 213), (3) GA estimates of below 27 weeks or above 44 weeks (n = 1,442), (4) mother’s age less than 15 years or greater than 44 years (n = 851), (5) presence of one or more identified congenital anomaly (n = 2,086), and (6) PTBs with a procedure code for induction of labor (n = 5,335).

Exposure to ambient air pollution during pregnancy was calculated using a previously developed gridded data fusion product at a 12-km spatial resolution.^30^ Specifically, numerical model simulations from the Community Multi-scale Air Quality Model (CMAQ) were bias-corrected with monitoring measurements in Georgia. Each birth was linked to a CMAQ grid cell based on the maternal address census block group at delivery. Exposures during the first and second trimester were obtained by averaging daily concentration estimates for five pollutants: 1-hour maximum carbon monoxide (CO) and nitrogen oxides (NOx); 24-hour average particulate matter less than 2.5 μm in aerodynamic diameter (PM_2.5_); and the PM_2.5_ components elemental carbon (EC) and organic carbon (OC). Trimester exposures were calculated separately based on either the clinical or LMP-based GA.

### Statistical analysis

We considered four different PTB definitions. A birth was designated as a PTB if (1) the LMP-based GA was <37 weeks, (2) the clinical GA was <37 weeks, (3) either the LMP-based or the clinical GA was <37 weeks, or (4) both the LMP-based and the clinical GA were <37 weeks. For PTB definitions (3) and (4), we used the average of trimester exposures calculated using conception dates estimated from LMP-based and clinical GA as the exposure.

We first analyzed how PTB outcome uncertainty varied across demographic variables and air pollution exposures. Among births diagnosed as PTB using either the clinical or the LMP-based GA, we defined a discordant indicator when these two PTB diagnoses differed. Using logistic regression, we first regressed the discordant indicator on a set of demographic covariates. Associations between discordance and exposures were evaluated one-at-a-time by adding air pollution exposure to the model with demographic covariates. We excluded concordant full-term births in this analysis to avoid comparing the subset of PTBs to a reference group dominated by full-term births.

For each PTB definition, we used logistic regression to estimate associations between pollutant exposures during the first and second trimesters and PTB. In the air pollution and PTB models, we adjusted for maternal education (less than 9th grade, 9th to 12th grade, high school graduate, college), race (Asian, black, Hispanic, white, other), tobacco use during pregnancy, residential county, a smooth function of poverty level as measured by block group–level percent below poverty, and a smooth function of estimated conception date. Smooth functions were parameterized using natural cubic splines with five and twelve degrees of freedom for poverty and conception date, respectively. Other variables including maternal age, alcohol use, and number of previous births were examined as potential confounders but did not impact the air pollution association estimates and were ultimately removed.

We also directly incorporated the additional uncertainty in the PTB definition using a multiple imputation approach. Binary PTB status was imputed through draws from a binomial distribution defined based on the two estimates of GA. Specifically, the probability of PTB, *P*, is defined as the proportion of weeks less than 37 among the GA range given by the clinical and the LMP-based estimates. For example, if the two GA estimates for a birth were 33 and 39 weeks, the probability of PTB is *P* = 4/7 (4 weeks of being PTB among seven total weeks). Concordant PTB status from LMP-based and clinical estimates of GA had *P* = 1 and concordant full-term births had *P* = 0. We took draws from the resulting binomial distributions for each birth to obtain 25 imputed data sets and performed separate logistic regressions to estimate air pollution associations with the aforementioned covariates for each set. The resulting 25 coefficient estimates and standard errors for pollutant effects were combined using the method by Rubin.^31^

## Results

The study cohort consisted of 267,801 singleton births from the 20-county Atlanta metropolitan area. Of these births, 8.31% (n = 22,262) were preterm using LMP estimates of GA; 7.40% (n = 19,828) were preterm using clinical estimates; 9.67% (n = 25,903) were preterm based on either the LMP or clinical determination; and 6.04% (n = 16,187) were preterm when there was concordance between LMP and clinical estimates. Hence, agreement in PTB diagnoses only occurred in 62.5% of PTBs identified using either LMP or clinical estimate of GA. Table 1 provides additional summary statistics of the study cohort characteristics stratified by preterm status. Supplementary Table S1; http://links.lww.com/EE/A23 provides summary statistics among PTBs based on the four definitions and shows negligible differences in maternal characteristics.

**Table 1:**
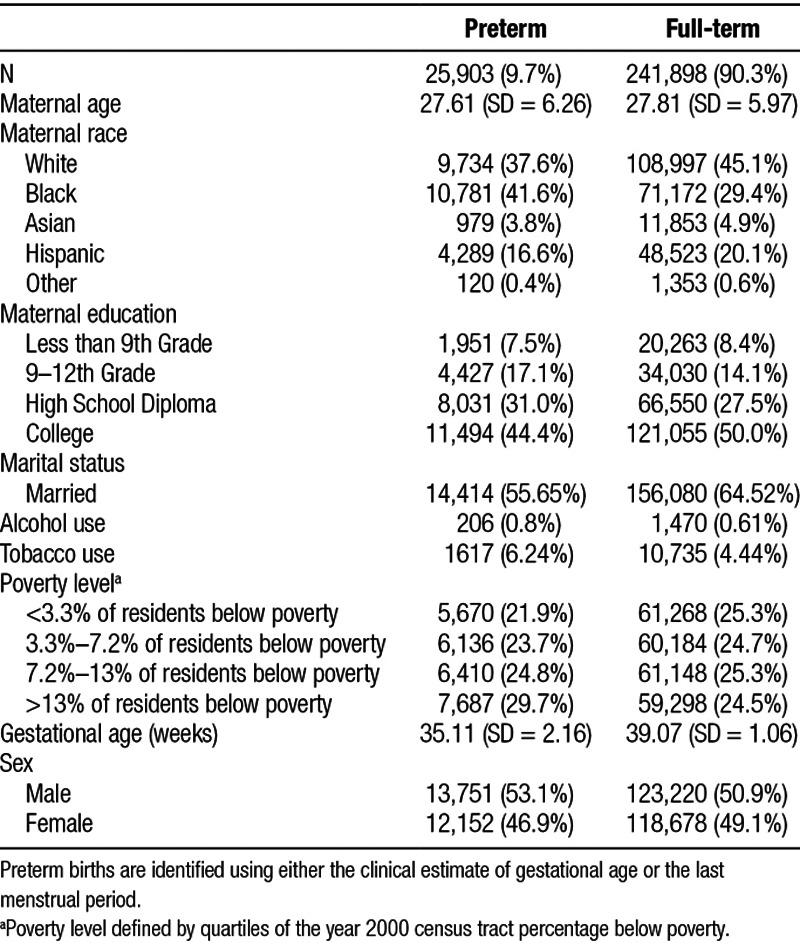
Maternal characteristics and demographics by preterm (gestational age <37 weeks) and full-term (gestational age ≥37 weeks) status of singleton births in the 20-county metropolitan Atlanta, Georgia, area from June 26, 2002, to December 16, 2006

Overall GA estimates were similar between LMP and clinical definitions, but larger disagreements occurred among PTBs. Among all births, 54.1% of GA estimates were identical; 32.4% of estimates differed by 1 week; 10.2% of estimates differed by 2 weeks; and 3.3% of estimates differed by 3 weeks or more. However, among births with either an LMP or clinical PTB diagnosis, only 39.9% of GA estimates were identical and 12.8% differed by 3 weeks or more.

Trimester-wide average pollutant exposures were similar across the three different assessment methods: using the conception date derived from LMP, clinical estimate, or an average of the previous two. Table 2 summarizes the mean exposure level for each pollutant and trimester, as well as the interquartile range (IQR) for the LMP definition. Correlations between exposures based on LMP and clinical estimates were very high, ranging from 0.976 to 0.999, indicating uncertainty in GA had minimal impacts on trimester-average exposures.

**Table 2:**
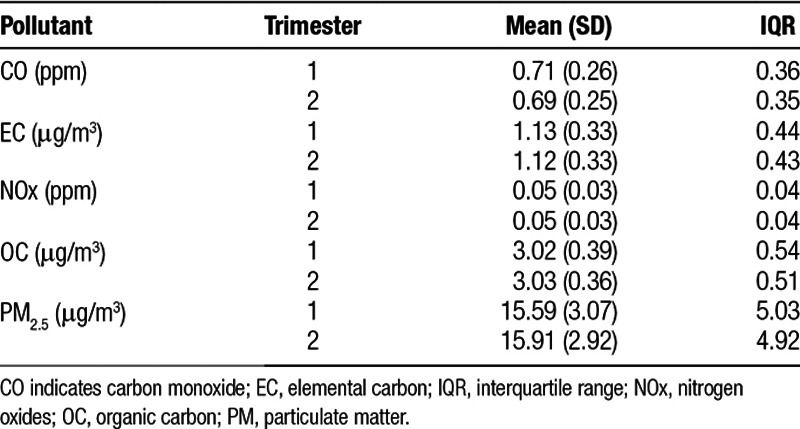
Summary statistics of gestational air pollutant exposures during the first and second trimester derived using LMP gestational age estimates.

Among births with at least one PTB diagnosis (either clinical or LMP), higher odds of disagreement between diagnoses was associated with maternal race/ethnicity (Hispanic versus non-Hispanic, Asian versus White, and White versus Black), married mothers, and tobacco use during pregnancy. Higher trimester-wide exposures to CO and NOx were associated with lower odds of disagreement. Specific odds ratios (ORs) and 95% confidence intervals for this disagreement analysis are given in Supplementary Table S2; http://links.lww.com/EE/A23.

**Figure 1.**
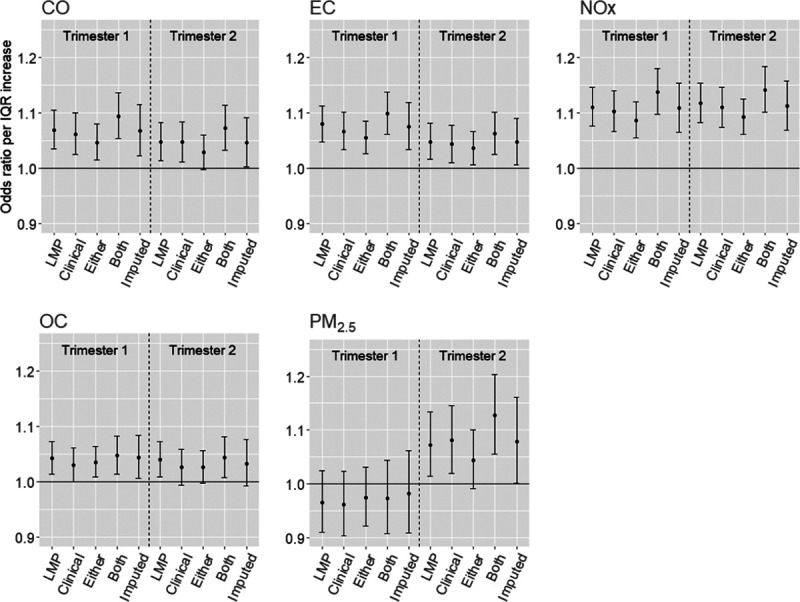
Estimated associations between preterm birth (PTB) and per interquartile range (IQR) increase in pollutant exposure during trimester 1 and 2. PTB is defined using the last menstrual period (LMP), the clinical estimate of gestational age, either LMP or clinical (either), both LMP and clinical agreement (both), and via imputation (imputed). CO indicates carbon monoxide; EC, elemental carbon; NOx, nitrogen oxides; OC, organic carbon; PM, particulate matter.

Figure 1 shows the estimated associations between PTB and average pollutant concentration during trimester 1 and trimester 2 using various PTB definitions. Log ORs and 95% confidence intervals for all exposure and PTB definition combinations are given in Supplementary Table S3; http://links.lww.com/EE/A23. Adjusting for demographic covariates and spatial-temporal trends, exposure to CO, EC, NOx, and OC during the first trimester was consistently associated with increased odds of PTB using all PTB definitions. CO, EC, NOx, OC, and PM_2.5_ exposures in the second trimester were associated with most PTB definitions. Second trimester exposure to NOx, on a per-IQR level, was most strongly associated with PTB.

Estimated ORs per IQR exposure using the clinical PTB definition are generally similar to estimates using the LMP PTB definition. Using the most stringent definition of PTB (agreeing diagnoses) consistently yielded the largest ORs. In contrast, ORs obtained from PTB defined using either clinical or LMP-based GA (i.e., least stringent definition) tended to be the lowest among the PTB definitions. For example, average PM_2.5_ during the second trimester was associated with ORs: LMP OR = 1.07, Clinical OR = 1.08, Either OR = 1.04, and Both OR = 1.13. Across pollutants and trimesters, differences in OR estimates for these two PTB definitions ranges from 0.0% to 8.3%. Similar patterns were observed in stratified analyses by maternal race (black versus non-black), maternal ethnicity (Hispanic versus non-Hispanic), and maternal marital status as shown in Supplementary Figures S1, S2, and S3; http://links.lww.com/EE/A23.

Using imputed PTB status gives point estimates that tend to be between estimates based on either LMP or clinical diagnoses and estimates based on agreeing diagnoses. More importantly, confidence intervals from the imputed estimates were between 7.4% and 43.8% wider than the other PTB definition estimates. Median increases in interval length across exposures are 30.1%, 25.1%, 10.6%, and 40.5% comparing imputed PTB status to LMP-based, clinical, both, or either PTB diagnosis, respectively.

## Discussion

We observed positive associations between several pollutants and PTB in both the first and second trimester using different PTB definitions. Using the most stringent definition of PTB (agreeing diagnoses) resulted in elevated associations, while using the least stringent definition of PTB (either diagnosis) resulted in the weakest associations. This observation may be attributed to increased sensitivity that may minimize outcome misclassification among true PTB, leading to less effect attenuation. It is also possible that the larger air pollution OR for the more stringent PTB definition is due to the lower baseline rates of PTB.

Uncertainty in GA can contribute to both outcome misclassification and exposure measurement error when timing of exposure during gestation is important. A previous study of PM_2.5_ and PTB by Rappazzo et al.^27^ found that substantially more births were classified as PTB using LMP estimates. This is consistent with our data. However, the degree of difference in estimated air pollution associations across different PTB definitions in our study was smaller. This may be because (1) we used trimester-wide averages while Rappazzo et al. used weekly averages, and (2) the comparison by Rappazzo et al. was carried out using two different cohorts because not all birth certificates contained both LMP and clinical GA estimates.

Previous studies had predominantly defined PTB using only the LMP-based GA or only the clinical GA. In our study, these two PTB definitions gave nearly identical OR estimates, suggesting that the choice of LMP-based or clinical GA for defining PTB may have limited impact on between-study heterogeneity. However, several factors have been suggested as potential contributors to the observed heterogeneity in studies of air pollution and birth outcomes.^6,7,32^ These include differences in particulate matter composition, the distributions of effect modifiers, residual confounding due to the use of various proxy measures of socioeconomic status, the magnitude of air quality measurement errors, and statistical methodologies.

Using a stringent definition of PTB (e.g., concordant LMP and clinical diagnoses), we may minimize true full-term births being classified as preterm, but some truly PTB will be classified as full term. However, we consider this pattern of misclassification preferable due to its increased specificity. In our study, more than 90% of births were classified as full term using any definition of PTB. Incorrectly classifying full-term births as preterm would have a large impact by diluting the smaller PTB group with full-term births. Conversely, incorrectly classifying PTBs as full term would have negligible impact due to the large number of full-term births.

We found that trimester-wide average exposures were not sensitive to the choice of PTB definition. Hence, GA uncertainty likely contributes minimal exposure measurement error relative to other sources such as maternal residential mobility^33, 34^ and spatiotemporal exposure modeling of air pollution concentration.^35^

We found several demographic variables (e.g., married versus unmarried mother, and maternal race White versus Black) to be associated with higher rate of discordant diagnoses. These associations may reflect differences in GA across subpopulations, where very preterm GA is likely to have fewer discordant diagnoses. For example, among births with at least one PTB diagnosis, the average LMP GA was 35.1 weeks for married mothers versus 34.8 weeks for unmarried mothers and 35.2 weeks for maternal race whites versus 34.7 weeks for maternal race blacks.

Even though the birth certificate provides two estimates of GA, the true GA cannot be ascertained given the retrospective nature of the study design. We hence implemented a multiple imputation approach to introduce uncertainty and variability associated with the estimated GA and, consequently, the PTB diagnosis. Multiple imputation has been utilized to address outcome misclassification when validation data are available to estimate sensitivity and specificity.^36^ Given the large study sample size, we found robust associations between air pollutant exposure and PTB in our analyses with imputation, despite increases in confidence interval widths. This result suggests that findings from previous studies may not be qualitatively different despite the presence of outcome misclassification.

Several additional issues regarding PTB misclassification warrant future investigations. First, our imputation model assumes that the true GA is between the clinical and LMP estimates from the birth records; the true GA may be outside this range. Second, we focused solely on the first and second trimester exposure where the exposure window has fixed length and is only referenced by the estimated conception date. For time-varying and short-term exposures, further methods development is needed in order to handle outcome misclassification when time-to-event models are used to analyze PTB or log-linear models are used to analyze time-series of PTB counts.

Our study does not call into question results from previous ambient air pollution and PTB research using either LMP or clinical birth record estimates of GA, although reported associations may be underestimated compared to those obtained using a more stringent definition of PTB. Furthermore, associations reported in previous studies are likely not due to outcome misclassification, based on our findings using a multiple imputation approach to incorporate uncertainty in PTB diagnosis. The impacts of PTB uncertainties should be further examined in other study regions and time periods. We encourage exploring different definitions of PTB when possible and recommend the use of a PTB definition based on both clinical and LMP-based criteria. While using an agreeing definition of clinical and LMP PTB determinations will reduce power due to a decreased number of cases, studies leveraging birth records can likely achieve sufficient sample size. In our study, we did not observe a substantial increase in standard error between the use of agreeing LMP and clinical definitions compared to using either LMP or clinical definition of PTB.

## Conflicts of interest statement

The authors declare that they have no conflicts of interest with regard to the content of this report.

## Supplementary Material
