# Supplementary material for: Impacts of gestational age uncertainty in estimating associations between preterm birth and ambient air pollution
Source: Environ Epidemiol. 2018 Dec 12;2(4):e031. doi: 10.1097/EE9.0000000000000031 (PMC7660973; doi:10.1097/EE9.0000000000000031)

Online Supplementary Materials

Impacts of gestational age uncertainty in estimating associations between preterm birth and ambient air pollution

Benjamin E Nealy[1], Joshua L Warren[2], Matthew J Strickland[3], Lyndsey A Darrow[3], Howard H Chang[1]

1. Department of Biostatistics and Bioinformatics, Emory University, 2. Department of Biostatistics, Yale University, 3. School of Community Health Sciences, University of Nevada, Reno

Corresponding author:
Howard H Chang
Department of Biostatistics and Bioinformatics
Emory University
1518 Clifton Rd, Atlanta, 30322
Phone: (404) 712-4627
howard.chang@emory.edu

Supplementary Table S1. Maternal characteristics and demographics among preterm births in the 20-county metropolitan Atlanta, Georgia area from 26 June 2002 to 16 December 2006. PTB is defined using the last menstrual period (LMP), the clinical estimate of gestational age, either LMP or clinical (either), both LMP and clinical agreement (both). The total number of births is 267,801.

		**LMP**	**Clinical**	**Both LMP and Clinical**	**Either LMP or Clinical**
**N**		22262 (8.31%)	19828 (7.40%)	16187 (6.04%)	25903 (9.7%)
**Maternal Age**		27.64 (6.28)	27.67 (6.28)	27.72 (6.33)	27.61 (SD = 6.26)
**Maternal Race**	White	8109 (36.43%)	7530 (37.98%)	5905 (36.48%)	9734 (37.6%)
	Black	9473 (42.55%)	8331 (42.02%)	7023 (43.39%)	10781 (41.6%)
	Asian	843 (3.79%)	725 (3.66%)	589 (3.64%)	979 (3.8%)
	Hispanic	3732 (16.76%)	3152 (15.90%)	2595 (16.03%)	4289 (16.6%)
	Other	105 (0.47%)	90 (0.45%)	75 (0.46%)	120 (0.4%)
**Maternal Education**	Less than 9th Grade	1717 (7.71%)	1416 (7.14%)	1182 (7.30%)	1951 (7.5%)
	9-12th Grade	3850 (17.29%)	3407 (17.18%)	2830 (17.48%)	4427 (17.1%)
	High School Diploma	6847 (30.76%)	6051 (30.52%)	4867 (30.07%)	8031 (31.0%)
	College	9848 (44.24%)	8954 (45.16%)	7308 (45.15%)	11494 (44.4%)
**Marital Status**	Married	54.82% (49.77%)	55.54% (49.69%)	54.38% (49.81%)	55.65% (49.7%)
**Alcohol Use**		0.8% (8.93%)	0.86% (9.22%)	0.88% (9.36%)	0.8% (8.9%)
**Tobacco Use**		6.38% (24.45%)	6.38% (24.44%)	6.6% (24.84%)	6.24% (24.2%)
**Poverty Level***	[0,0.033)	4867 (21.86%)	4405 (22.22%)	3602 (22.25%)	5670 (21.9%)
	[0.033,0.0722)	5234 (23.51%)	4679 (23.60%)	3777 (23.33%)	6136 (23.7%)
	[0.0722,0.13)	5414 (24.32%)	4904 (24.73%)	3908 (24.14%)	6410 (24.8%)
	[0.13,1.01)	6747 (30.31%)	5840 (29.45%)	4900 (30.27%)	7687 (29.7%)
**Gestational Age (weeks)**		34.49 (SD = 2.06)	34.51 (SD = 2.12)	34.15 (SD = 2.18)	35.11 (SD = 2.16)
**Sex**	Male	11858 (53.27%)	10529 (53.10%)	8636 (53.35%)	13751 (53.1%)
	Female	10404 (46.73%)	9299 (46.90%	7551 (46.65%)	12152 (46.9%)

Supplementary Table S2: Estimated odds ratios (OR) and 95% confidence interval (CI) between preterm birth (PTB) discordance, and maternal demographic characteristics and air pollution exposures. Discordance was defined as different clinical and last menstrual period (LMP) determinations of PTB among all PTB births diagnosed using either the clinical or the LMP-based gestational age estimate.

	**OR [95% CI]**
Alcohol use during pregnancy	0.888 (0.655, 1.204)
Tobacco use during pregnancy	**0.8 (0.713, 0.897)**
Maternal ethnicity: Hispanic versus non-Hispanic	**1.108 (1.008, 1.217)**
Maternal race: Asian versus White	**1.152 (1.003, 1.324)**
Maternal race: Black versus White	**0.895 (0.833, 0.96)**
Maternal race: Other versus White	0.985 (0.676, 1.435)
Maternal education: 9th-12th grade versus < 9th grade	0.922 (0.816, 1.041)
Maternal education: High School versus < 9th grade	1.043 (0.927, 1.174)
Maternal education: College versus < 9th grade	0.955 (0.843, 1.081)
% Poverty: [3.3%, 7.2%) versus < 3.3%	1.08 (0.999, 1.167)
% Poverty: [7.2%, 13%) versus < 3.3%	1.067 (0.985, 1.156)
% Poverty: > 13% versus < 3.3%	0.997 (0.915, 1.087)
Infant sex: Female versus Male	1.031 (0.98, 1.085)
Unmarried versus Married	**0.881 (0.826, 0.939)**
Maternal age: (19,24] versus ≤ 19	1.095 (0.994, 1.206)
Maternal age: (24,29] versus ≤ 19	1.039 (0.938, 1.15)
Maternal age: (29,34] versus ≤ 19	0.987 (0.887, 1.099)
Maternal age: (34,39] versus ≤ 19	0.895 (0.794, 1.007)
Maternal age: (39,44] versus ≤ 19	**0.822 (0.684, 0.988)**
CO Trimester 1**	**0.896 (0.849, 0.945)**
CO Trimester 2**	**0.908 (0.861, 0.958)**
EC Trimester 1**	0.972 (0.929, 1.017)
EC Trimester 2**	0.967 (0.923, 1.013)
NOx Trimester 1**	**0.913 (0.869, 0.959)**
NOx Trimester 2**	**0.926 (0.881, 0.974)**
OC Trimester 1**	0.984 (0.946, 1.023)
OC Trimester 2**	1.005 (0.966, 1.044)
PM2.5 Trimester 1**	1.001 (0.96, 1.044)
PM2.5 Trimester 2**	0.967 (0.926, 1.01)

* Estimates are controlled for county-specific indicator variables. ** Pollutant exposure coefficients are scaled by the interquartile ranges (IQR) given below:

**Pollutant**	**IQR**
CO Trimester 1 (ppm)	0.368
CO Trimester 2 (ppm)	0.355
EC Trimester 1 (μg/m^3)	0.443
EC Trimester 2 (μg/m^3)	0.444
NOx Trimester 1 (ppm)	0.039
NOx Trimester 2 (ppm)	0.038
OC Trimester 1 (μg/m^3)	0.532
OC Trimester 2 (μg/m^3)	0.499
PM2.5 Trimester 1 (μg/m^3)	4.962
PM2.5 Trimester 2 (μg/m^3)	4.951

Supplementary Table S2: Estimated associations between preterm birth (PTB) and per interquartile range (IQR) increase in pollutant exposure during trimester 1 and 2. PTB is defined using the last menstrual period (LMP), the clinical estimate of gestational age (Clinical), either LMP or clinical (Either), or both LMP and clinical (Both). For PTB defined using either or both gestational age estimates, trimester exposures are the average of exposures derived using LMP-based or clinical estimates of conception date.

Pollutant	Trimester	PTB Definition	Exposure	Log OR per IQR	95% Confidence Interval
**CO**	1	Both	Average	0.0896	[0.0442, 0.1349]
	1	Either	Average	0.0450	[0.0080, 0.0821]
	1	LMP	LMP	0.0664	[0.0270, 0.1058]
	1	Clinical	Clinical	0.0595	[0.0180, 0.1010]
	2	Both	Average	0.0697	[0.0249, 0.1146]
	2	Either	Average	0.0274	[-0.0092, 0.0640]
	2	LMP	LMP	0.0455	[0.0064, 0.0846]
	2	Clinical	Clinical	0.0455	[0.0044, 0.0866]
**EC**	1	Both	Average	0.0935	[0.0522, 0.1348]
	1	Either	Average	0.0534	[0.0199, 0.0870]
	1	LMP	LMP	0.0764	[0.0405, 0.1122]
	1	Clinical	Clinical	0.0642	[0.0267, 0.1016]
	2	Both	Average	0.0602	[0.0179, 0.1024]
	2	Either	Average	0.0351	[0.0007, 0.0696]
	2	LMP	LMP	0.0464	[0.0095, 0.0833]
	2	Clinical	Clinical	0.0420	[0.0035, 0.0805]
**NOx**	1	Both	Average	0.1286	[0.0852, 0.1720]
	1	Either	Average	0.0827	[0.0474, 0.1181]
	1	LMP	LMP	0.1044	[0.0668, 0.1420]
	1	Clinical	Clinical	0.0971	[0.0575, 0.1367]
	2	Both	Average	0.1321	[0.0891, 0.1751]
	2	Either	Average	0.0882	[0.0532, 0.1232]
	2	LMP	LMP	0.1107	[0.0732, 0.1481]
	2	Clinical	Clinical	0.1035	[0.0642, 0.1428]
**OC**	1	Both	Average	0.0461	[0.0071, 0.0852]
	1	Either	Average	0.0351	[0.0035, 0.0667]
	1	LMP	LMP	0.0421	[0.0087, 0.0755]
	1	Clinical	Clinical	0.0301	[-0.0050, 0.0652]
	2	Both	Average	0.0432	[0.0010, 0.0854]
	2	Either	Average	0.0263	[-0.0079, 0.0605]
	2	LMP	LMP	0.0393	[0.0028, 0.0758]
	2	Clinical	Clinical	0.0258	[-0.0122, 0.0639]
**$PM_{2.5}$**	1	Both	Average	-0.02696	[-0.1101, 0.0562]
	1	Either	Average	-0.02615	[-0.0933, 0.0410]
	1	LMP	LMP	-0.03558	[-0.1065, 0.0353]
	1	Clinical	Clinical	-0.03975	[-0.1141, 0.0346]
	2	Both	Average	0.11975	[0.0417, 0.1978]
	2	Either	Average	0.04323	[-0.0197, 0.1061]
	2	LMP	LMP	0.06996	[0.0030, 0.1369]
	2	Clinical	Clinical	0.07764	[0.0079, 0.1474]

Supplementary Figure S1. Estimated associations, stratified by maternal race (Black versus non-Black) between preterm birth (PTB) and per interquartile range (IQR) increase in pollutant exposure during trimester 1 and 2. PTB is defined using the last menstrual period (LMP), the clinical estimate of gestational age, either LMP or clinical (either), both LMP and clinical agreement (both), and via imputation (imputed).

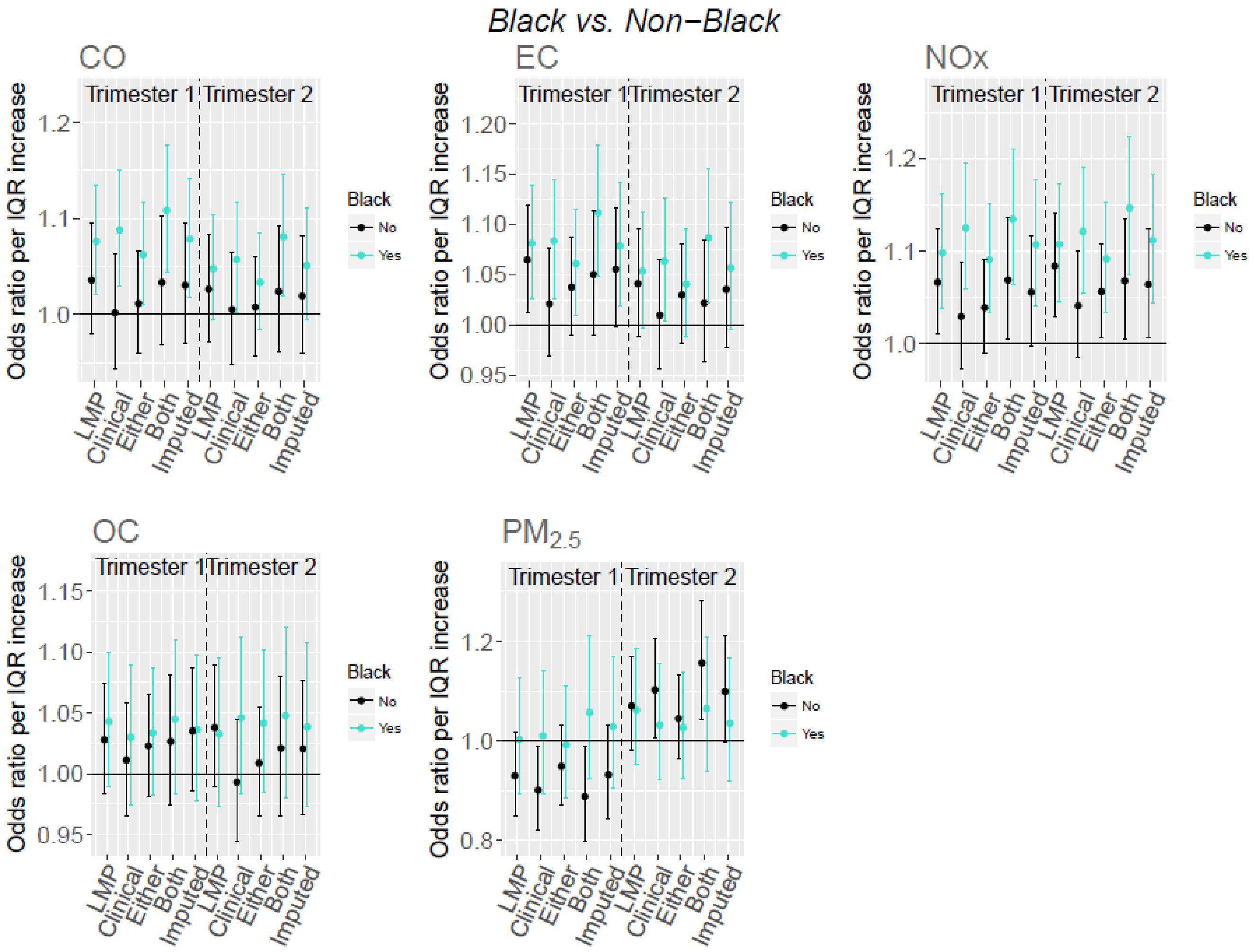

Supplementary Figure S2. Estimated associations, stratified by maternal ethnicity (Hispanic versus non-Hispanic) between preterm birth (PTB) and per interquartile range (IQR) increase in pollutant exposure during trimester 1 and 2. PTB is defined using the last menstrual period (LMP), the clinical estimate of gestational age, either LMP or clinical (either), both LMP and clinical agreement (both), and via imputation (imputed).

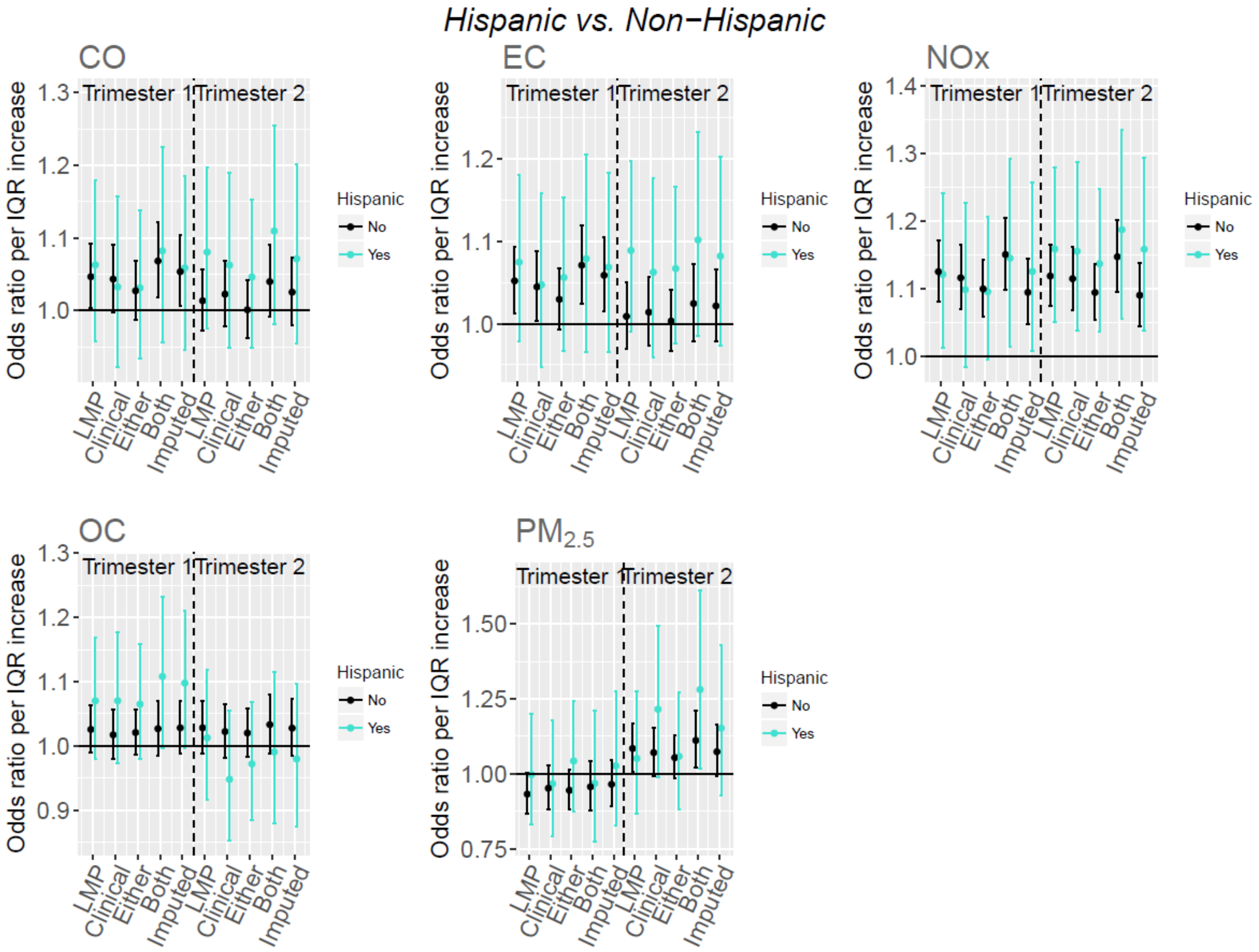

Supplementary Figure S3. Estimated associations, stratified by maternal marital status (married versus unmarried) between preterm birth (PTB) and per interquartile range (IQR) increase in pollutant exposure during trimester 1 and 2. PTB is defined using the last menstrual period (LMP), the clinical estimate of gestational age, either LMP or clinical (either), both LMP and clinical agreement (both), and via imputation (imputed).

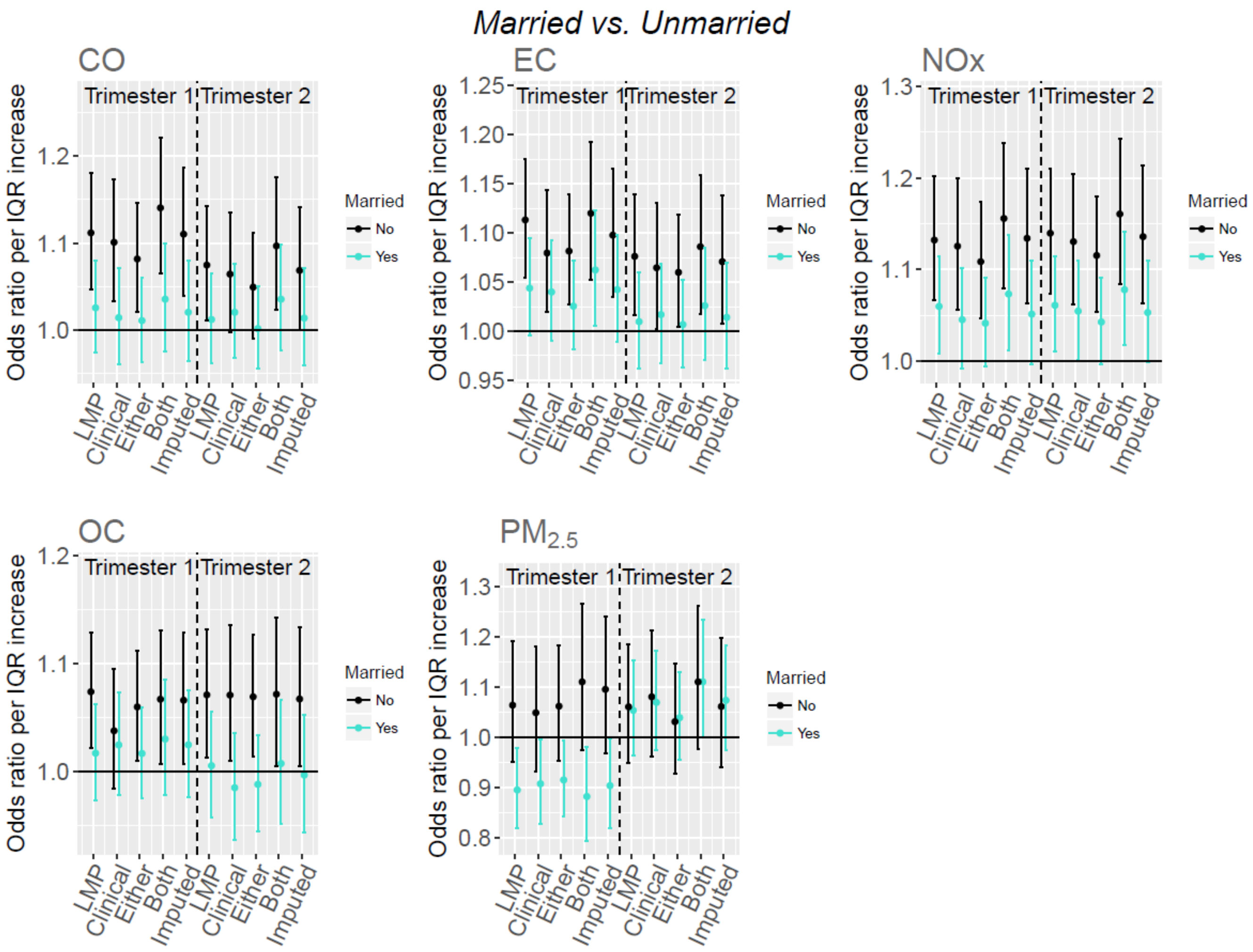

Supplement: Supplementary file 1 [file ee9-2-e031-s001.pdf]
